# Noninvasive assessment of age-related stiffness of crystalline lenses in a rabbit model using ultrasound elastography

**DOI:** 10.1186/s12938-018-0509-1

**Published:** 2018-06-13

**Authors:** Xinyu Zhang, Qingmin Wang, Zhen Lyu, Xuehua Gao, Pengpeng Zhang, Haoming Lin, Yanrong Guo, Tianfu Wang, Siping Chen, Xin Chen

**Affiliations:** 1 School of Biomedical Engineering, Health Science Center, Shenzhen University, Shenzhen, China; 2 National-Regional Key Technology Engineering Laboratory for Medical Ultrasound, Shenzhen, China; 3 Guangdong Key Laboratory for Biomedical Measurements and Ultrasound Imaging, Shenzhen, China

**Keywords:** Crystalline lens, Stiffness, Ultrasound elastography, Acoustic radiation force, Shear wave, Group velocity

## Abstract

**Background:**

The pathological or physiological changes of a crystalline lens directly affect the eye accommodation and transmittance, and then they increase the risk of presbyopia and cataracts for people in the middle and old age groups. There is no universally accepted quantitative method to measure the lens' mechanical properties in vivo so far. This study aims to investigate the possibility of assessing the age-related stiffness change of crystalline lens by acoustic-radiation-force-based ultrasound elastography (ARF-USE) in a rabbit model in vivo.

**Methods:**

There were 13 New Zealand white rabbits that were divided into four groups and fed normally until they were 60 (n = 4), 90 (n = 2), 120 (n = 4), and 150 (n = 3) days old, respectively. An ARF-USE platform was built based on the Verasonics™ Vantage 256 system. The shear waves were excited and traced in the lens by a linear ultrasound probe after a rabbit was anaesthetized.

**Results:**

The average group velocities were 1.38 ± 0.2 m/s, 2.06 ± 0.3 m/s, 2.07 ± 0.29 m/s, and 2.30 ± 0.28 m/s, respectively, for the four groups of rabbits. The results shows that the group velocity has a strong correlation with the day age (r = 0.84, p < 1 × 10^−7^) and the weight (r = 0.83, p < 1×10^−7^) of the rabbits while the maximum displacement has no correlations with the day age (r = 0.27, p > 0.1) and the weight (r = 0.32, p > 0.1).

**Conclusion:**

This study demonstrated that the group velocity measured by ARF-USE had a strong correlation with age-related stiffness in a rabbit model, suggesting that group velocity is a good biomarker to characterize the stiffness of a crystalline lens. This study also demonstrated the feasibility of using this USE technique to assess the mechanical properties of the lens in vivo for clinical or research purposes.

## Background

Crystalline lens is a nearly transparent biconvex structure that is suspended behind the iris of the eye. As an important component of the eye’s refractive system, it is responsible for focusing the light rays coming from the target onto the retina. The lens has three main parts: the capsule, the epithelium, and fibers. The lens capsule forms the outermost layer of the lens and the lens fibers form the bulk of the interior of the lens. The cells of the lens epithelium are located between the lens capsule and the outermost layer of the lens fibers and they are found only on the anterior side of the lens. Lens fiber production continues throughout life, with the new fibers being laid down on the outer side of the older fibers; the growth results in concentric layers of secondary lens fibers, which form an onion-like structure. The lens has three functions: to maintain its own clarity, to refract light, and to provide accommodation. Both the optical and mechanical properties are of great importance to vision quality. A cataract occurs and leads to poor vision when the clarity progressively degrades. The lens is the only component that has the accommodation ability in the eye. Patients with presbyopia feel that it is hard to focus clearly on close objects with the gradual loss of accommodation. The pathological or physiological changes of the crystalline lens directly affect the eye accommodation and transmittance, and then they increase the risk of presbyopia and cataract. This risk rises remarkably with age [1], which seriously influences elderly patients’ quality of living and poses a huge public health burden. There are currently no other effective treatments—except surgical replacement and wearing glasses—and there is limited knowledge about the pathogenesis of lens diseases. No definite method has been developed to delay the progress of symptoms or reverse age-related changes of the lens.

Many ex vivo experiments have been designed to investigate the mechanical properties of the crystalline lens in past few decades, including spinning tests [2], mechanical tensile tests [3, 4], compression tests [5], indentation tests [1], dynamic mechanical tests [6, 7], and microbubble displacement tests [8, 9]. While these experiments have increased our general knowledge about the mechanical properties of the lens in animals and humans, there is no universally accepted quantitative method to measure the lens’ mechanical properties in vivo so far. Without in vivo methods, it is hard to know the individual differences in mechanical properties, the pathological or physiological alteration, and the effectiveness of a certain therapy. Some researchers have been working on in vivo methods. Scarcelli et al. proposed a method of assessing the lens’ stiffness with Brillouin microscopy in 2011 [10, 11]. They performed Brillouin scans along the crystalline lens in 56 eyes from 30 healthy subjects aged from 19 to 63 years in 2016 [12] and found that the adult human lens showed no measurable age-related increase in the peak longitudinal modulus while the central stiff plateau region expands steadily over age from 19 to 63 years. The low imaging speed of this technique is still a shortcoming for its wide application in clinics. Wu et al. suggested a measurement system that pushed the lens with ultrasound (US) acoustic radiation force (ARF) and recorded the vibration with an optical coherence tomography (OCT) system in 2015 [13]. This system can image wave propagation with high resolution, but it needs to align the focus of US and OCT. Ultrasound elastography (USE), as a means for the in vivo measurement of mechanical properties, has made rapid progress in the past two decades. There are several representative techniques, including quasi-static elastography, transient elastography (TE) [14], sonoelastography [15], acoustic radiation force impulse (ARFI) imaging elastography [16], supersonic shear wave imaging (SSI) [17], shear wave elasticity imaging (SWEI) [18], and shear wave dispersion ultrasound vibrometry (SDUV) [19]. Among these techniques, ARFI, SSI, SWEI, and SDUV use ARF to excite the tissue to vibrate and are known as acoustic-radiation-force-based USE (ARF-USE). ARF-USE has a special advantage for assessing the lens’ stiffness since it can palpate the tissue remotely and detect the vibration using a single ultrasound probe. Detorkis et al. applied the Aixplorer^®^ system (SuperSonic Imagine, Aix-en-Provence, France) in 2014 to evaluate the pilocarpine and atropine instillation on the lens’ stiffness in rabbits’ eyes [20], which demonstrated the possible use of USE for in vivo measurement. Park et al. investigated the effect of intraocular pressure (IOP) on elastic wave velocity in 2016 [21] and found that the dependency of velocity on IOP was significantly lower in comparison with that of cornea in assessing the stiffness using USE technique.

The motivation of this study is to investigate an in vivo method for assessing the mechanical properties of the lens in a non-destructive manner. It may be helpful to understand the pathogenesis of lens diseases more deeply and to promote the development of new treatments. We measured the group velocity of the ARF-induced shear wave in the rabbit lens in vivo using an USE research platform, and we performed a correlation study to investigate the possibility of an in vivo assessment of age-related stiffness change for crystalline lenses in a rabbit model using ARF-USE.

## Methods

### Experimental systems

**Fig. 1:**
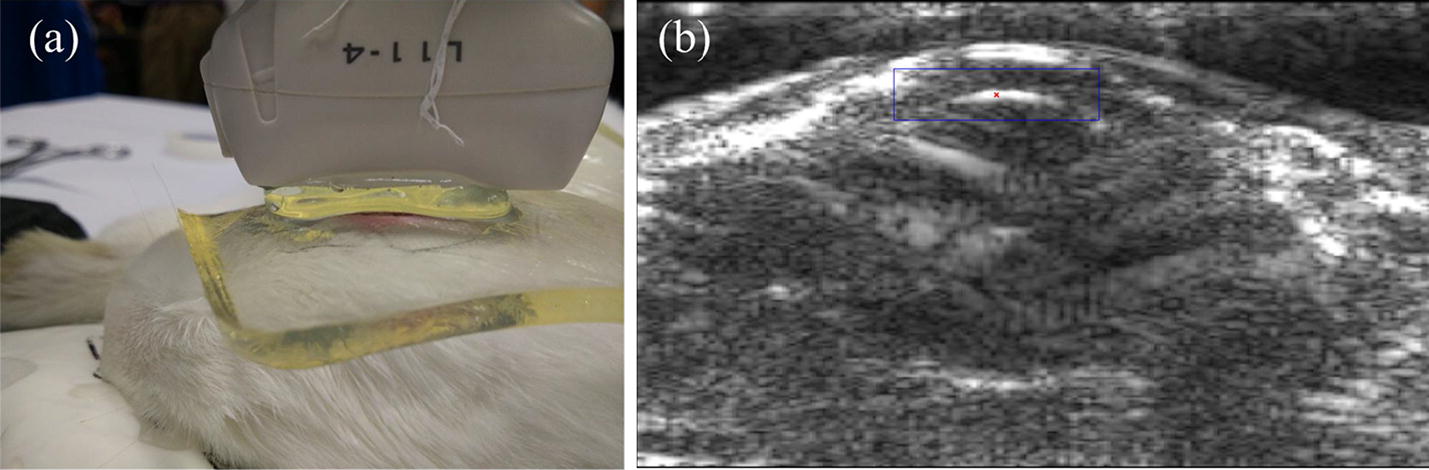
Measurement of lens: **a** Experimental measurement setup; and **b** a B-mode ultrasound image

An experimental platform was constructed based on the Verasonics™ Vantage 256 system with a linear array probe (L11-4) of 128 elements (Fig. 1a). The Verasonics system is a flexible hardware and software platform which provides a direct access to the raw channel data from each element of the array in real time as well as a software beamformer to form an ultrasound image. It has been widely adopted by research labs to conduct ultrasound research. A good tutorial was given in [22] for details about the implementation of shear wave elasticity imaging using a Verasonics system. The programming script and the data processing method used in this study were similar to those described in that tutorial.

**Fig. 2:**
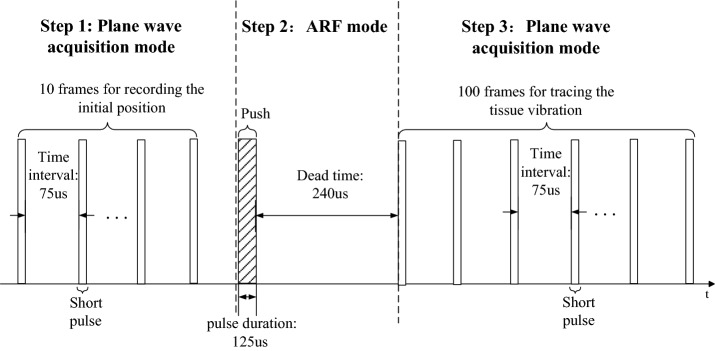
A diagram of complete pulse sequence

We implemented both B-mode imaging and USE measurement on this platform. The B-mode image in Fig. 1b was presented to the operator to display the anatomical cross-section of the target. After the operator selected the measurement point of stiffness evaluation, the system was switched to a USE mode for stiffness evaluation. The excitation and detection sequence of this mode is illustrated in Fig. 2. The whole sequence was divided into three steps. First, the system worked in the plane wave acquisition mode and acquired 10 frames of ultrasound place wave image to record the initial position of the measurement point. Second, the system worked in the ARF mode and ARF was generated to push the lens for 125 μs and then slept for 240 μs. Finally, the system acquired 100 frames of plane wave image to trace the tissue vibration. The time interval between the frames here was 75 μs, which means that the system traced the tissue vibration using 13.3 kHz temporal sampling frequency. In the ARF mode, the focused ARF was generated by 35 focused array elements, emitting 500 cycles of 4 MHz sine wave with the maximum excitation voltage of 58 V. In the plane wave acquisition mode, 128 channels simultaneously transmitted short pulses with a center frequency of 6.25 MHz and received an echo signal. The maximum voltage was 70 V and the pulse repetition frequency was 13.3 kHz in this mode.

**Fig. 3:**
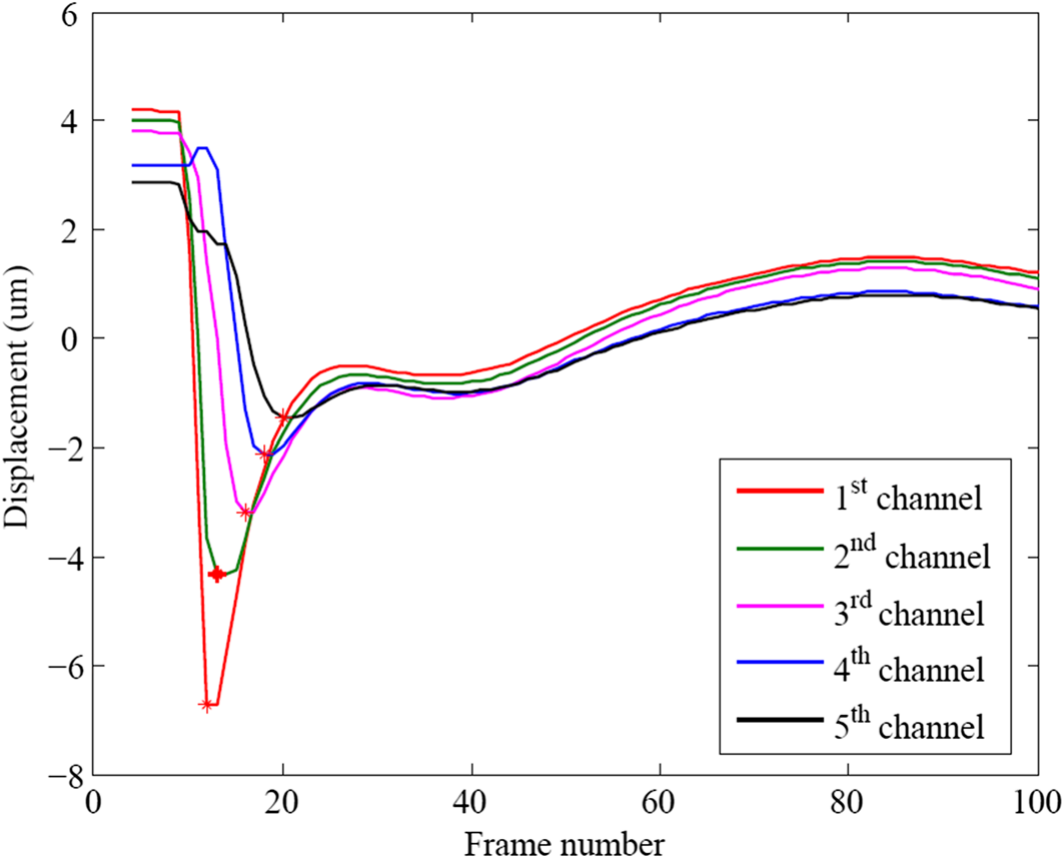
Local displacement waveforms extracted from the first five detection channels showing the propagating shear waves in the lens

The consecutive echo signals captured in step 3 (Fig. 2) were used to estimate the displacement of tissue by KANSI algorithm [22], a kind of phase difference detection method [23], which is basically the same method as that used in color Doppler and tissue Doppler. Figure 3 provides an example of five displacement waveforms obtained from five adjacent detection channels, each spaced 0.33 mm. The horizontal ordinate is the number of the frame, which refers to the temporal sampling points of the waveform. The group velocity refers to the velocity with which the envelope of the wave amplitudes propagates along the direction parallel with the probe. The wave arrival time was determined from displacement waveform using the time of peak displacement and then group velocity was estimated by the slope of linear regression between wave arrival time and lateral position [24].

Young’s modulus E was estimated based on the group velocity *c* of the induced shear wave as follows [25]:

1 $$ {\text{E}} = 3\rho {\text{c}}^{2} $$

where c is the group velocity of the shear wave measured by our platform and $\rho$ is the density of the tissue (1.1 × 10^3^ kg/m^3^) [26].

### Experimental design

We purchased 13 New Zealand white rabbits from the Guangdong Experimental Animal Center (Guangdong Province, China). They were divided into four groups and fed normally until they were 60, 90, 120, and 150 days old, respectively, in the Experimental Animal Center, Shenzhen University, China. During the experiment, the rabbits were weighted and then anesthetized by ear vein injection with 20% urethane (5 ml/kg). The rabbits’ eyelid reflex was checked to make sure the anesthetization had taken effect. The rabbits were moved to the experimental platform and kept in the lateral position. The eyelashes were glued to the eyelid with medical adhesive tape to avoid discomfort to the rabbits and interrupting the imaging. A liner-array probe (L11-4) was placed above one eye during the experiment. A self-made gelatin pad of 0.5-cm thickness as well as a coupling gel was placed between the eye and the probe to increase the coupling of the ultrasound. Measurements were obtained after the front segment of an eye was easily discerned in the B-mode image. With the guidance of the B-mode image, the operator chose a point near the apex of the lens and switch to USE mode for stiffness measurement. The tissue displacement waveform together with the maximum amplitude and the group velocity were calculated for each measurement. The average value of 12 measurements was determined for each eye. A Tukey test, in conjunction with analysis of variance (ANOVA), was used to test the significance of difference between two groups. Correlation analysis was performed to investigate the correlation of the maximum amplitude or group velocity with day ages of the rabbits.

## Results

**Fig. 4:**
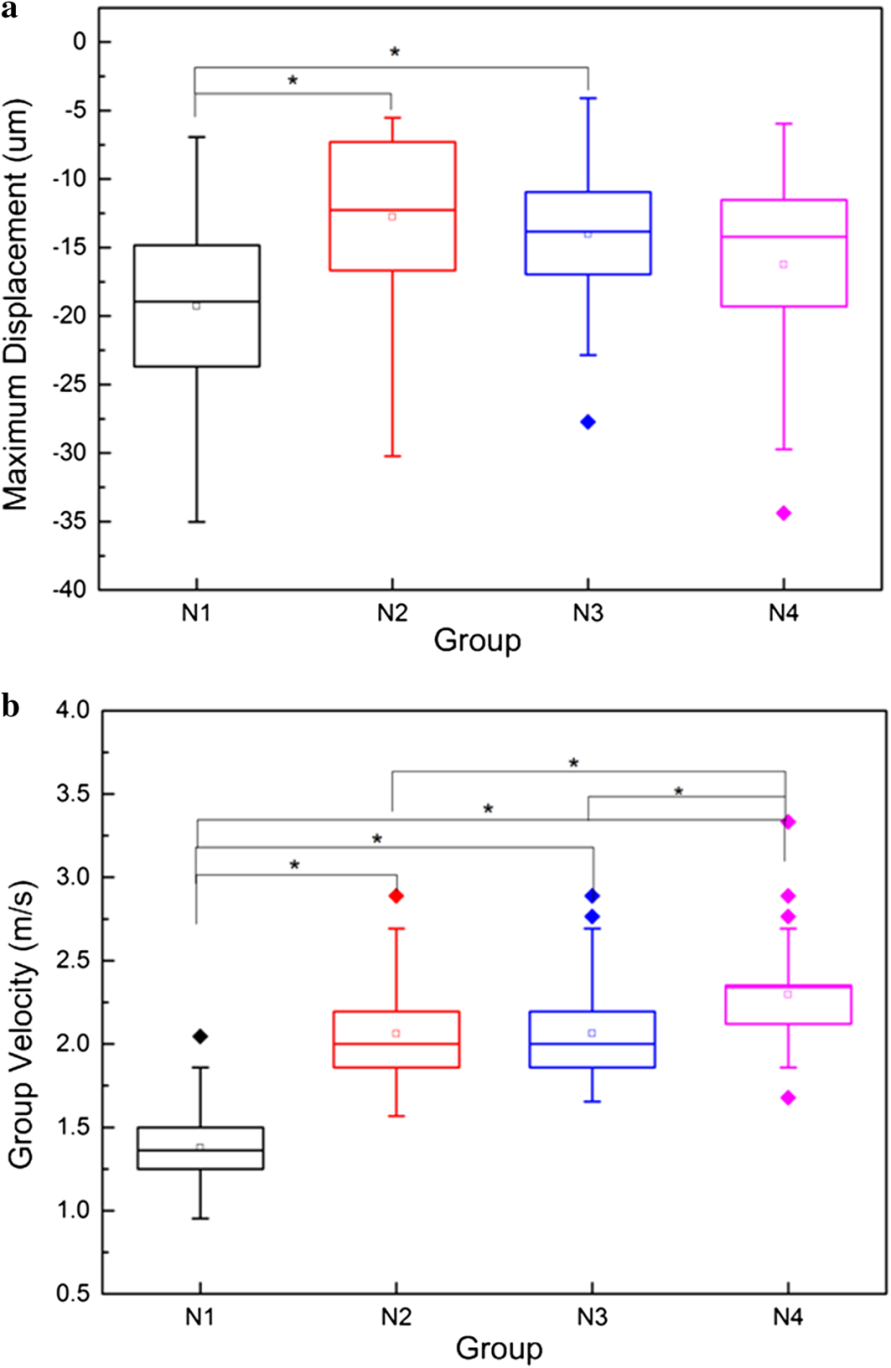
Box-line plot of: **a** maximum displacement; and **b** group velocity for group N1 (60 days), N2 (90 days), N3 (120 days), and N4 (150 days). Asterisk indicates that the mean values of the two groups are significantly different (p < 0.001)

The rabbits were divided into four groups. Groups N1, N2, N3, and N4 included four rabbits that were 60 days old, two that were 90 days old, four that were 120 days old, and three that were 150 days old, respectively. Each eye was measured separately for every rabbit. The group average of weight, maximum displacement, group velocity, and estimated Young’s modulus are listed in Table 1. A box-line plot of maximum displacement and group velocity for each group is provided in Fig. 4. One-way ANOVA indicated that the maximum displacements of the N1 group were obviously larger than those of the N2 and N3 groups (p < 0.001), and there were no significant differences among the groups. However, there were significant differences (p < 0.001) in the group velocities between any of two groups—except N2 and N3—which suggests that the group velocity can differentiate the variations in stiffness caused by 30 days in young rabbits.

**Table 1:** Measurement results of each group (average ± standard deviation)

Groups	Day ages (day)	Weight (kg)	Maximum displacement (μm)	Group velocity (m/s)	Young’s modulus (kPa)
N1 (n = 4)	60	1.7 ± 0.20	19.26 ± 6.14	1.38 ± 0.2	5.82 ± 1.75
N2 (n = 2)	90	2.25 ± 0.21	14.12 ± 5.88	2.06 ± 0.3	13.00 ± 4.01
N3 (n = 4)	120	2.69 ± 0.21	14.02 ± 4.78	2.07 ± 0.29	13.03 ± 3.90
N4 (n = 3)	150	2.82 ± 0.16	16.23 ± 6.80	2.30 ± 0.28	16.07 ± 4.05

**Fig. 5:**
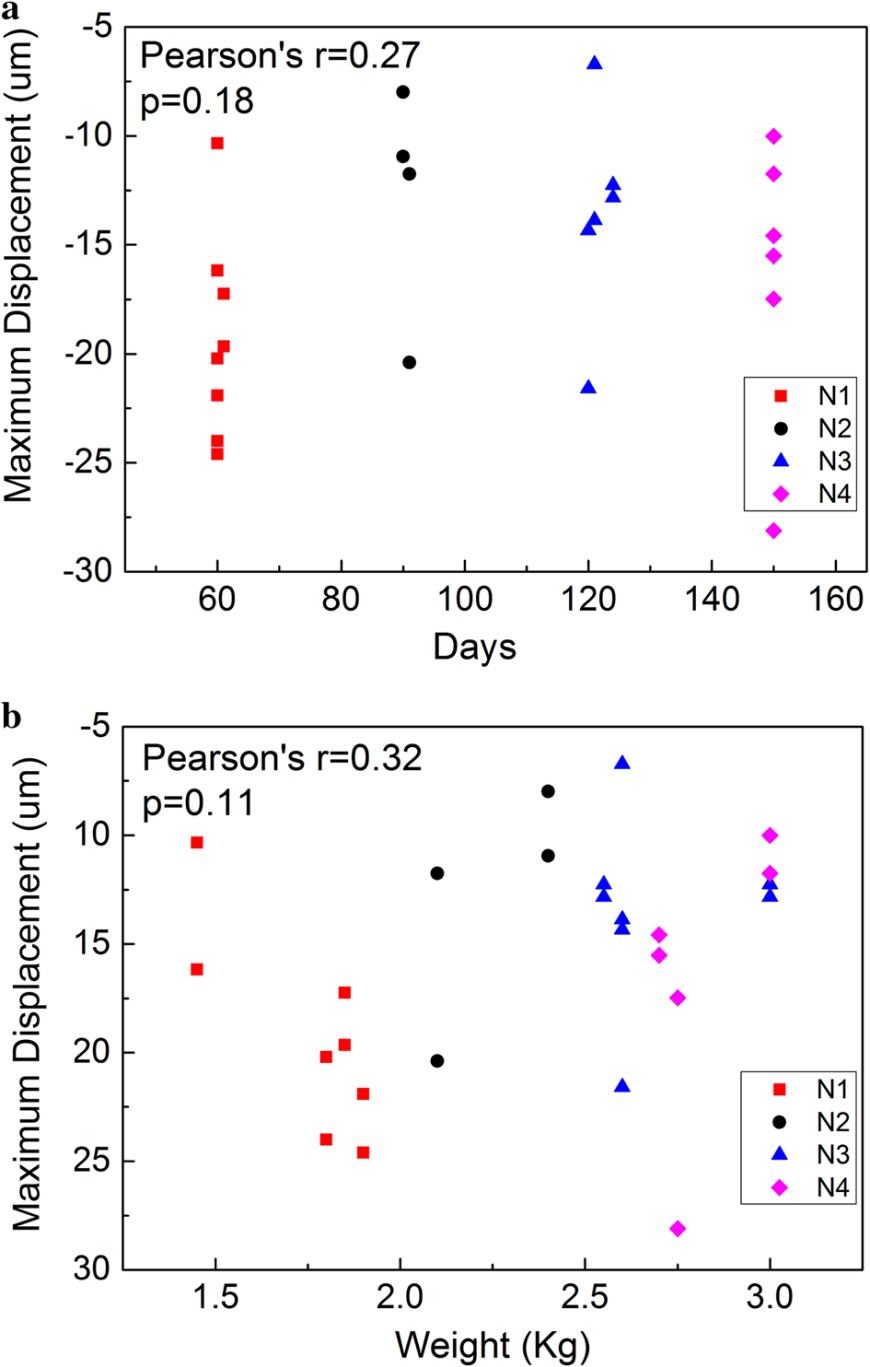
Correlation analysis of maximum displacement in rabbit crystalline lens with **a** day age and **b** weight. In total, there are 26 data points in a plot, and the Y-axis value of a point refers to the mean value of all of the measurements performed on one eye

**Fig. 6:**
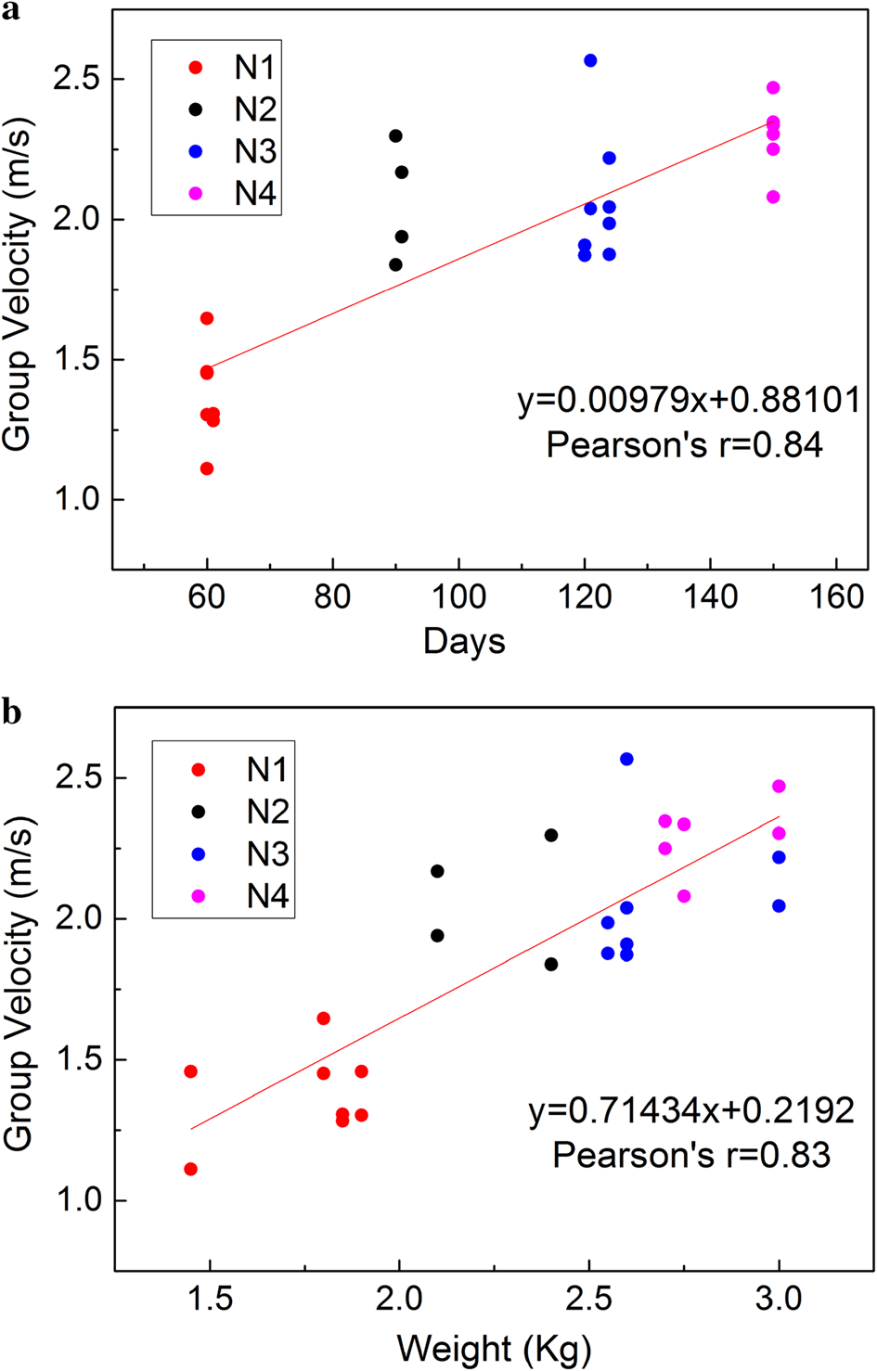
Correlation analysis of the group velocity in rabbit crystalline lens with **a** day age and **b** weight

As shown in Fig. 5, the correlation analysis found that the maximum displacement had no correlations with the day age (r = 0.27, p > 0.1) and with the weight (r = 0.32, p > 0.1). However, in Fig. 6, the group velocity had a strong correlation with the day age (r = 0.84, p < 1 × 10^−7^) and the weight (r = 0.83, p < 1 × 10^−7^).

## Discussion

It is well known that lens stiffness has a strong correlation with age [10, 27–31]. Pau et al. used a miniature dynamometer to measure the penetration resistance of the fine conical probe to different lens layers to investigate the relationship between human lens stiffness and age [1]. They found that the penetration resistance of the lens increased with age in patients who were 20–84 years old. Glasser et al., who performed mechanical compression tests in 19 freshly obtained lenses from cadaver ranging in age from 5 to 96 years [32], found that human lens stiffness had an exponential growth with age and that lens stiffness increased more than four-fold for a person from birth to death in 2001. Heys et al. employed a dynamic mechanical analyzer fitted with a custom-made probe to measure lens stiffness in different ages in 2004 [6], and the results showed that the logarithmic shear modulus of both the nuclear and cortical tissue in a lens increased with age in patients ranging from 14 to 78 years old. Furthermore, the increasing amplitude of nuclear tissue was most pronounced. Weeber et al. measured the local dynamic stiffness in 10 human lenses ranging from 10 to 78 years [33], and they found that the center stiffness and the periphery stiffness of the lenses had different increasing rates with age in 2007. Wu et al. combined US ARF with an OCT system to measure the low-amplitude elastic deformation with high resolution in 2015 [13]. Their results showed that the elastic deformation of young rabbit lenses was greater than that of mature rabbit lenses, which also suggested that lenses got stiffer when the rabbits grew up. All of the above studies showed that the stiffness of crystalline lens increased with age both in rabbits and humans and this was a physiological change. This study was carried out to confirm if it was possible to use the group velocity, a parameter measured by the USE technique, to assess the changes in the stiffness of the lens. Since there are few methods to alter the mechanical properties of the lenses in vivo, we used rabbits of different ages as an animal model to obtain various levels of lens stiffness for measurement.

The results showed that, in young rabbits (younger than 150 days old), the group velocity changed remarkably with the increase of the day age. The results showed significant differences (p < 0.001) in the group velocities between the N1 and N2 groups as well as between the N3 and N4 groups. It implies that by using the group velocity as a parameter, we can differentiate the changes in lens stiffness caused by a 30-day increase of age in young rabbits. The estimated Young’s moduli are 5.82 ± 1.75 kPa for the N1 group (60 days) and 16.07 ± 4.05 kPa for the N4 group (150 days), which is about three times that of the N1 group. The estimated Young’s moduli are nearly in the same order of magnitude in comparison with the findings of Wu et al. who evaluated the stiffness of rabbit lenses with a system combining an OCT with US in 2015 [13]. The strong correlation between the group velocity and the day age demonstrates that the group velocity may be a good biomarker to assess the stiffness of the lenses. The maximum displacement has no correlation with the day age, suggesting that it is not a reliable biomarker to characterize the stiffness of crystalline lens.

This work is a preliminary study that utilizes USE to characterize the stiffness of crystalline lens. The findings show that group velocity can reflect the physiological changes in the stiffness of rabbit lens. This study demonstrates the possibility for establishing an ultrasound-based technique to evaluate the mechanical properties of lens quantitatively in a noninvasive manner in vivo for clinical or research purposes. USE has a special advantage in measuring the mechanical properties of the lens in that the measurements can be performed by a single US system. The pushing and tracking can be carried out by a single transducer. It may become an easy-to-use method to monitor the stiffness of lenses in physiological or pathological conditions, which will be helpful in developing new treatments.

This study has several limitations that need to be addressed in future study. First, the number of subjects was limited so the conclusions lack strong statistical meanings. As shown in Fig. 4b, there is no significant difference on group velocity between group N2 and N3. It is hard to make a conclusion if this is caused by the actual physiological condition or measurement error due to small number of subjects. And the changes in stiffness were examined in a relatively short term (from 60 to 150 days). Future work will be carried out on a larger number of subjects and over a longer period of time. Meanwhile, factors that may affect the results need to been carefully examined. Park et al showed that the group velocity in lens had weak dependence on IOP in bovine eyeballs ex vivo but this result may be not conclusive for rabbit and human case in vivo [21]. Due to the ability of accommodation, different light condition can change the form of the lens, which may lead to the change in mechanical properties. Second, future work will also focus on improving the system performance in sensitivity and accuracy by using high-frequency ultrasound probe and advanced signal processing algorithm. With higher temporal resolution, the velocity of the elastic wave can be measured more accurately. Similarly, with higher detection sensitivity, small displacements can be easily detected, and less power of radiation force is needed. FDA recommendations of ultrasound power safety required that spatial-peak-time-average intensity (I_spta_) was less than 17 mW/cm^2^ for ophthalmic application, which was much lower than that for general application. Tanter et al. performed a stiffness measurement on porcine cornea by SSI technique and reported that I_spta_ was as low as 2.33 mW/cm^2^ if the measurement sequence, including ARF push and plane wave acquisition, was performed once in a second [34], which implied that, with optimized system and sequencing, it was possible for AFR-USE to meet the power safety requirement and be used in clinical ophthalmic applications. Finally, we used a very simple equation to estimate the Young’s modulus. Considering that the lens was small and round in shape and the pushing was applied on the surface, this simple equation may not precisely describe the relationship between the Young’s modulus and the elastic wave velocity. A future study will investigate a better model to estimate the Young’s modulus and it will be validated with classical mechanical testing.

## Conclusion

This study demonstrated that the group velocity measured by USE showed a strong correlation with the age-related stiffness in a rabbit model and suggested that the group velocity was a good biomarker to characterize the stiffness of a crystalline lens. This study also demonstrated the feasibility of using the USE technique to assess the mechanical properties of the lens in vivo, which will have great potential in studying the pathogenesis, diagnosis, and therapeutic treatment of presbyopia and cataracts in clinical ophthalmology.
